# Diagnostic Challenges and Recovery of First Tarsometatarsal Joint Tuberculosis Extending to Medial Cuneiform and First Metatarsal: A Rare Case Report

**DOI:** 10.1002/ccr3.70799

**Published:** 2025-08-20

**Authors:** Dahal Sweekar, Bist Omkar, Khati Sriram, Bhatta Sunil, Upadhyaya Maya, Pant Shubham

**Affiliations:** ^1^ Lalbandi Municipality Hospital Lalbandi Nepal; ^2^ Department of Orthopedics Nisarga Hospital and Research Center Kathmandu Nepal; ^3^ Department of Anesthesia Nisarga Hospital and Research Center Kathmandu Nepal; ^4^ Infectious and Communicable Disease Hospital Lekhnath Nepal; ^5^ Seti Provincial Hospital Dhangadhi Nepal

**Keywords:** anti‐tubercular therapy, diagnostic challenges, foot tuberculosis, rare occurrence, two‐stage surgery

## Abstract

Foot tuberculosis is rare and often diagnosed late due to non‐specific symptoms and lack of typical radiological findings. Early diagnosis and a combination of anti‐tubercular therapy and surgical intervention are crucial for preventing deformities, ensuring successful recovery without significant long‐term complications.

## 1 Introduction

Tuberculosis (TB) has afflicted humankind for millennia. The English writer John Bunyan referred to TB as “The Captain of these men of death” during a time when its prevalence in London was 1000 cases per 100,000 people per year [1]. Tuberculosis is a bacterial infection caused by *Mycobacterium tuberculosis* , first identified by Robert Koch in 1882 [1].

Even today, the global burden of tuberculosis remains significant. In 2023, an estimated 10.8 million people worldwide developed tuberculosis, 6.1% of which is in individuals living with HIV. In Nepal alone, there were approximately 68,000 reported cases [2]. While pulmonary TB accounts for the majority of cases, tuberculosis can affect almost any organ, including bones and joints [3]. Extrapulmonary TB constitutes about 10% of all cases, with skeletal TB making up approximately 3% of these. However, tuberculosis of the foot is exceptionally rare, comprising only 0.1%–0.3% of cases [4]. The talus and calcaneus are the most affected bones, followed by the midtarsal region, Lisfranc joints, and the ankle [5].

Here, we present the case of a 30‐year‐old man with right first tarsometatarsal joint tuberculosis extending to medial cuneiform and first metatarsal base who experienced a prolonged diagnostic delay and recovery. Initially, he was misdiagnosed as a case of gouty arthritis and pyogenic abscess at different health facilities. In addition, he also underwent incision and drainage before arriving at a definitive diagnosis. The time from symptom onset to diagnosis was 16 months. The rarity of this condition, the challenges in diagnosis, and the successful recovery through a combination of medical and surgical management—without any significant deformity—make this case particularly unique.

## 2 Case Presentation

### 2.1 History

Our patient, a 30‐year‐old man with no significant medical or surgical history, visited the general practitioner with complaints of dull, aching pain on the medial aspect of his right midfoot. The pain was persistent throughout the day and worsened with walking. Initially, he ignored the symptoms, but as the pain persisted, he decided to seek medical attention.

During his first visit, tenderness was noted on the medial aspect of the foot, near the right first tarsometatarsal joint. Investigations revealed mild hyperuricemia, for which he was prescribed a nonsteroidal anti‐inflammatory drug (NSAID), anti‐gout medication, and short‐term oral steroid. Following this, he reported slight pain relief. However, once he stopped taking analgesics, the pain gradually increased in intensity.

Over time, his pain progressively worsened. Four months after his initial consultation, he developed a red, painful swelling over the medial aspect of his right foot, corresponding to the site of maximum pain. On his second visit to a healthcare provider, an incision and drainage procedure was performed, revealing blood‐tinged discharge. He was discharged with oral antibiotics. However, after the procedure, he experienced chronic, intermittent discharge from the wound. Seeking further treatment, he visited another healthcare center, where a second incision and drainage was performed, leading to a temporary reduction in swelling. He was once again prescribed oral antibiotics.

Despite achieving normal serum uric acid levels and undergoing two incision and drainage procedures with two completed courses of oral antibiotics, he continued to experience pain and persistent discharge from the medial aspect of his right foot. This prompted him to consult an orthopedic specialist for further evaluation.

### 2.2 Investigations

Upon arrival at our outpatient department (OPD), he presented with pain, swelling, and discharge from the dorsal aspect of his right midfoot. There was no history of fever, night sweats, and weight loss.

Based on the presenting complaints, past medical history, and review of previous laboratory results, chronic osteomyelitis, cold abscess, musculoskeletal tuberculosis, and underlying gouty arthritis were considered as differential diagnoses. Additionally, the possibilities of malignancy and mycetoma were also taken into account. With these considerations, laboratory and radiological evaluations were carried out.

Laboratory investigations revealed:

- CBC: Hemoglobin: 14.5 g/dL, TLC: 9400/mm^3^, platelets: 420,000/mm^3^.
- Erythrocyte sedimentation rate (ESR): 35 mm/h.
- C‐reactive protein (CRP): 21.4 mg/L.
- Serum uric acid: 5.6 mg/dL.
- HIV status: Negative.

An ultrasonographic study showed cortical irregularities involving the medial cuneiform and first metatarsal, along with a hypoechoic area containing low‐level internal echoes, suggesting an active sinus with tenosynovitis of the tibialis anterior and extensor digitorum longus tendons.

**FIGURE 1:**
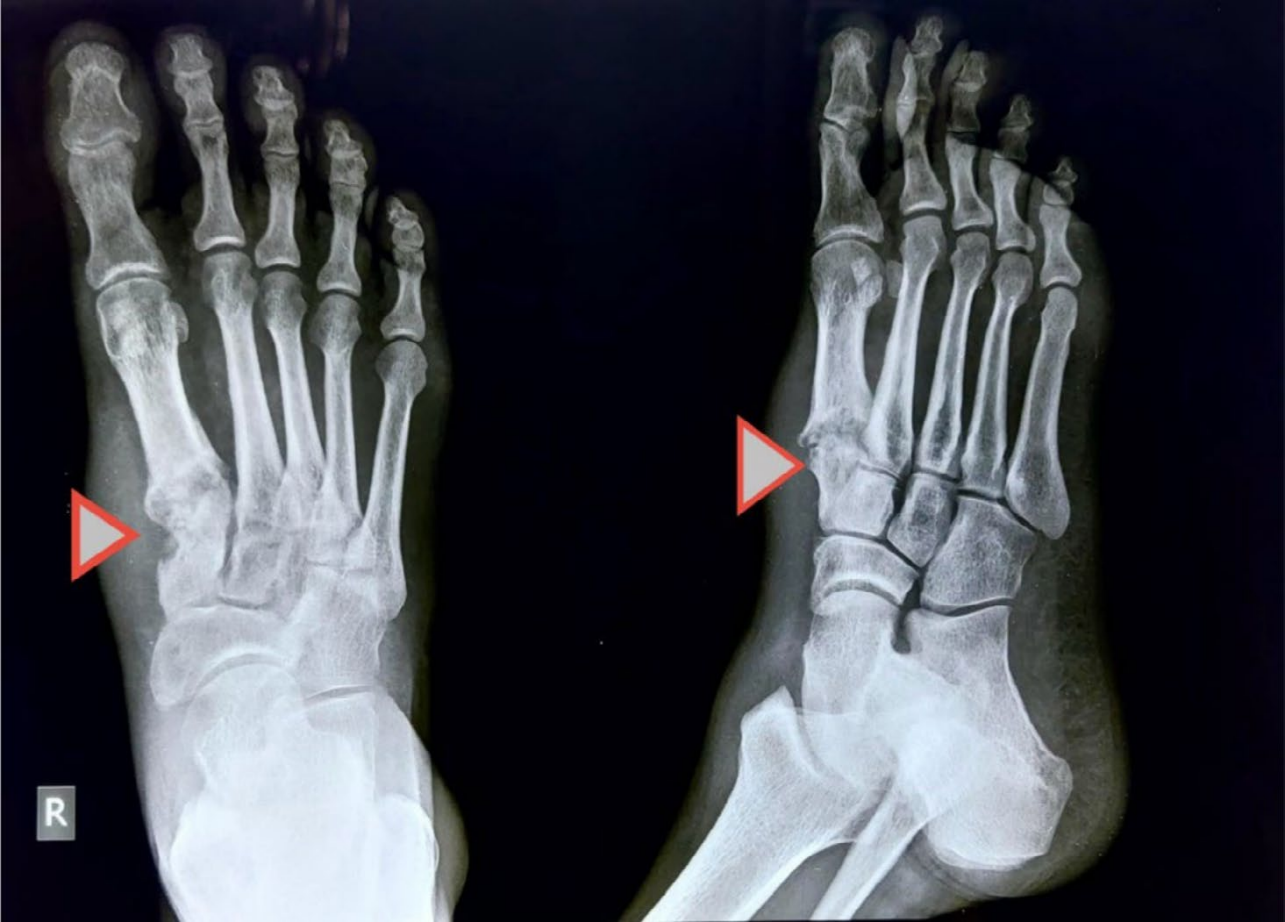
Arrowhead shows lytic lesion in medial cuneiform and base of 1st metatarsal.

An X‐ray of the right foot revealed cortical destruction of the medial cuneiform and the base of the first tarsometatarsal joint (Figure 1); chest X‐ray was normal.

Pus culture from the discharging sinus grew *Staphylococcus aureus* , which was sensitive to ceftriaxone, cefixime, amikacin, and levofloxacin among other antibiotics.

### 2.3 Diagnosis and Treatment

**FIGURE 2:**
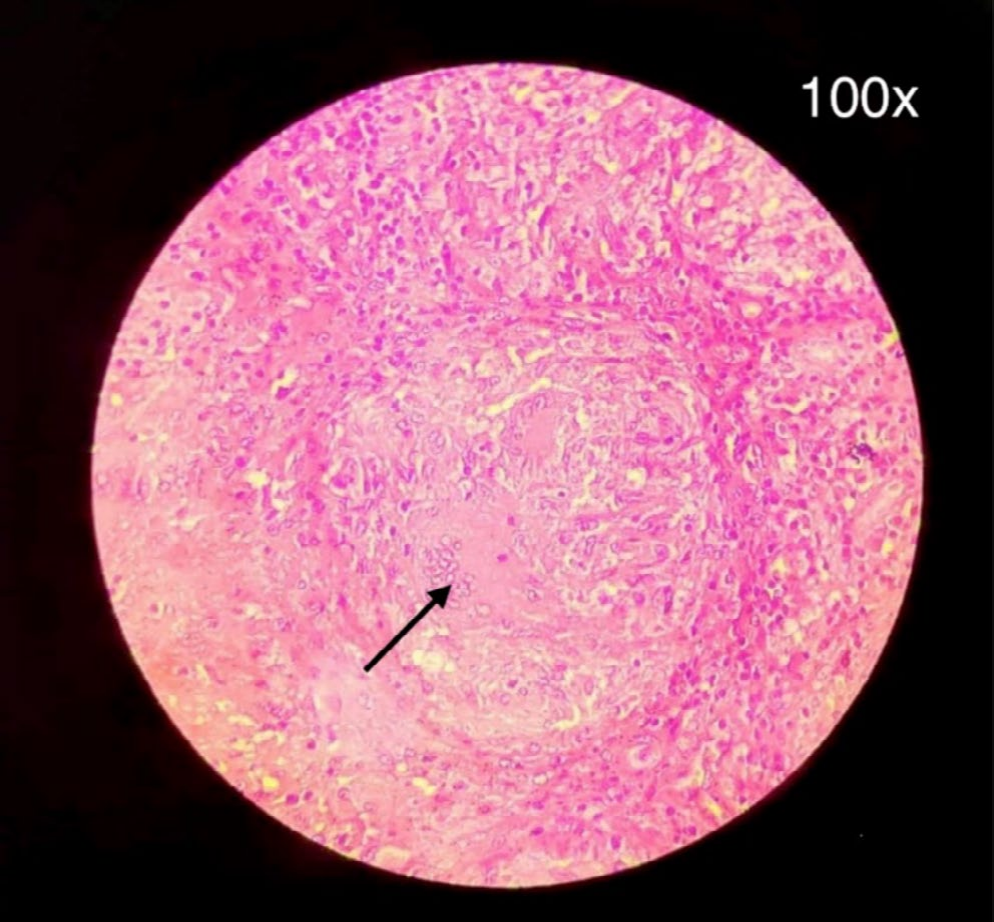
Caseating granuloma demonstrated from biopsy with arrow pointing to area of caseous necrosis.

Based on these findings, a provisional diagnosis of chronic osteomyelitis was made; surgical intervention was planned. Saucerization of the infected bone was performed, and vancomycin‐coated bone cement was implanted. A biopsy of the surgical specimen revealed a caseating granulomatous lesion, with epithelioid cell granulomas admixed with Langhans‐type giant cells, but no acid‐fast bacilli (AFB) staining (Figure 2).

Considering the clinical examination, investigations, and biopsy findings, a final diagnosis of tubercular arthritis of the right first tarsometatarsal joint with extension to the medial cuneiform and base of the first metatarsal with secondary pyogenic infection was made.

The patient was started on intravenous ceftriaxone 1 g twice daily and amikacin 750 mg (mg) once daily for two weeks, along with anti‐tubercular therapy (ATT). After two weeks of intravenous treatment, oral cefixime 200 mg twice daily and oral levofloxacin 750 mg once daily were continued for an additional four weeks. The selection of these antibiotics was guided by the drug sensitivity pattern, and the duration of therapy was determined based on standard treatment protocols, as well as clinical and radiological response. He was placed on a two‐month intensive phase of ATT, consisting of isoniazid 5 mg/kg, rifampicin 10 mg/kg, pyrazinamide 25 mg/kg, and ethambutol 15 mg/kg. Standard ATT therapy was administered according to the fixed drug combination regimen based on body weight, as recommended by the WHO. A below‐knee slab was applied, and non‐weight‐bearing crutch mobilization was recommended for 4 weeks to ensure immobilization.

**FIGURE 3:**
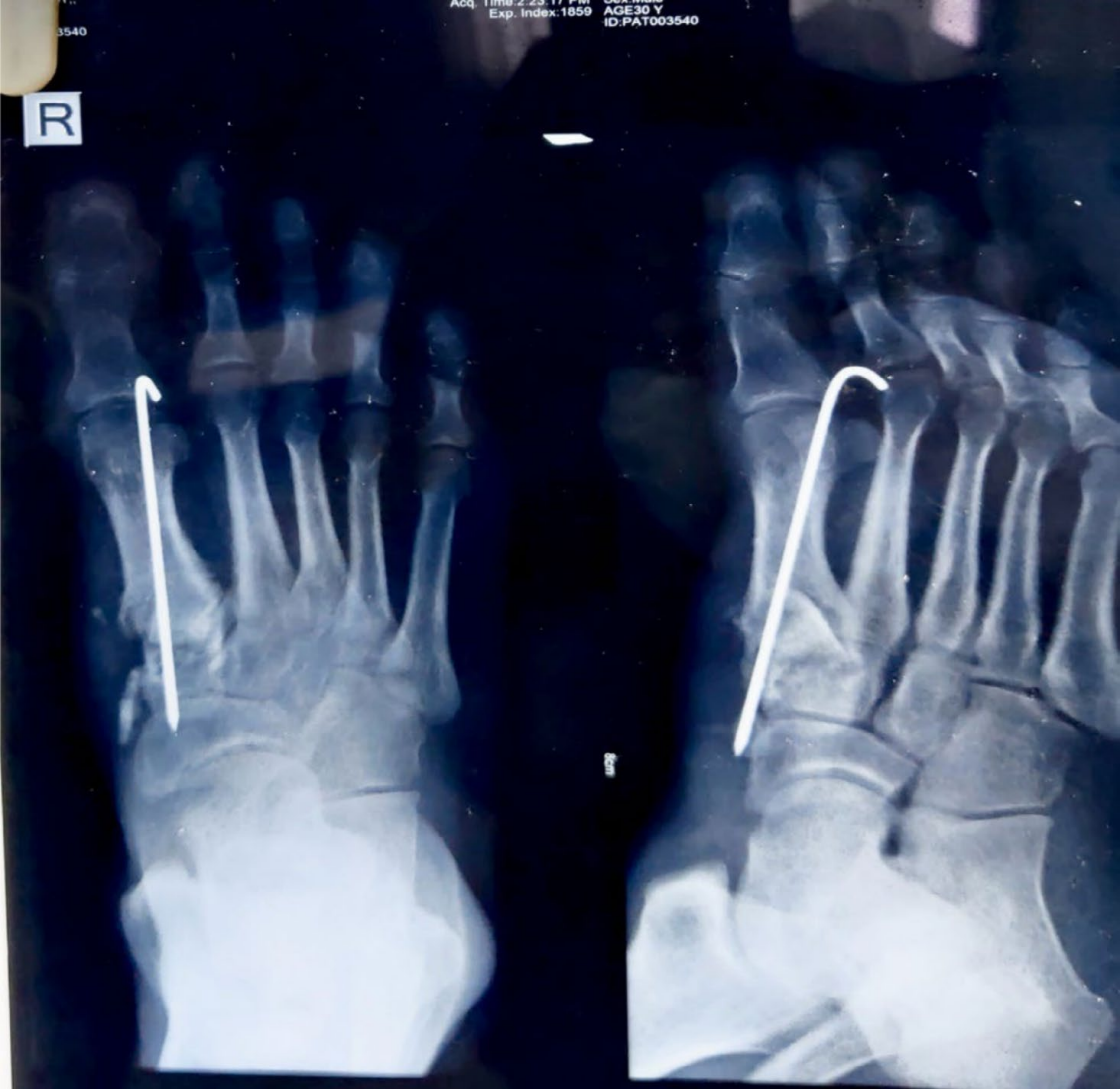
Bone grafting and K wire fixation of the affected site.

During his six‐week follow‐up, all wounds had healed completely, and there was no further discharge from the wound site. The bone cement was removed two months after implantation using the same incision. Simultaneously, a tricortical bone graft was harvested from the iliac crest and fixed with Kirschner wires (Figure 3). His ATT regimen was modified to the continuation phase, comprising isoniazid, rifampicin, and ethambutol, with the same previous dosage. The patient developed an acneiform rash, for which dermatological consultation was obtained. Anti‐tubercular therapy was pointed out as the culprit for the rash. With topical medication, the rash subsided. The patient also developed mild hyperbilirubinemia (2.1 mg/dL) for which physician consultation was done. ATT was continued as per the physician's recommendation. Hyperbilirubinemia subsided on the next follow‐up.

Due to extensive bone destruction, a slab was applied for four weeks, ensuring the complete rest of the affected foot. Partial weight‐bearing was permitted after four weeks, and K‐wires were removed at 12 weeks. By 12 weeks, the patient was allowed to bear full weight. Repeat X‐rays showed well‐healed bone with no significant deformities.

The patient continued with three‐monthly follow‐ups; ATT was discontinued after 18 months.

### 2.4 Outcome

**FIGURE 4:**
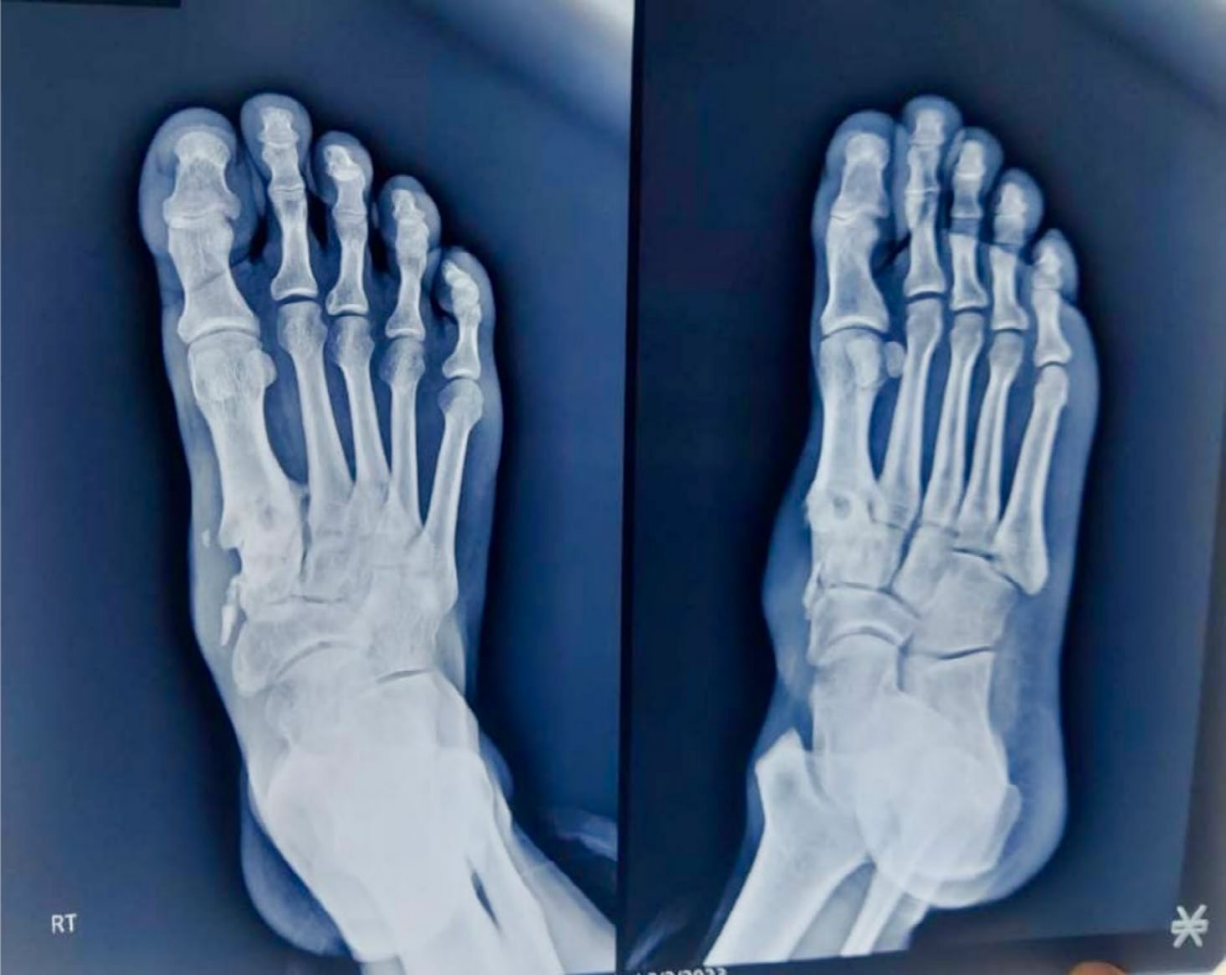
Follow up X‐ray at 18 months showing considerable healing of medial cuneiform and base of 1st metatarsal.

Subsequent laboratory tests remained within normal limits, and X‐rays confirmed complete bone healing without deformities (Figure 4). The patient is now pain‐free, with a normal gait and normal range of motion of his foot. He is able to bear full weight and has returned to his work without any limitations.

## 3 Discussion

Tuberculosis is a disease with immense global burden. Globally, 10.8 million people contracted tuberculosis in 2023 alone. This fits into a pattern following the COVID‐19 pandemic where there has been a gradual increase in the number of cases annually, whereas there was a prolonged steady decline for the past decade before the pandemic [2]. This is evident by the fact that there were 10.6 million cases in 2022, 10.3 million cases in 2021, and 10.0 million cases in 2020. Between 2015 and 2023, there has been a 15% reduction in incidence rate and an 11% reduction in mortality in Nepal. However, the End TB Strategy milestones are a 50% reduction in incidence and a 75% reduction in mortality in 2025 compared to 2015 [2]. This clearly shows it is unrealistic for Nepal to meet the milestones for 2025.

Tuberculosis primarily affects the lungs. But it can affect any organ, including the bones and joints [3]. As much as 30% of people with extra‐pulmonary TB show signs of concomitant pulmonary tuberculosis [6]. The spine is the most affected in musculoskeletal tuberculosis, owing to roughly half of the total cases. Foot and ankle tuberculosis are the rarer forms of skeletal TB [3, 6].

TB of the bone commonly results from hematogenous spread, usually originating from a primary infection site. The metaphysis receives the highest blood supply in the bone, making it the most frequent initial site of infection. The infection causes endarteritis and destruction of the epiphyses, leading to its spread into the joint space. If left untreated, cold abscesses may form around the bone and joints, often resulting in sinus tract formation. *Mycobacterium tuberculosis* does not produce cartilage‐destroying enzymes, and the absence of periosteal reaction or sclerosis is a characteristic pathological feature of tuberculous osteomyelitis [3].

Tuberculosis of the foot progresses slowly, often presenting with vague symptoms that resemble gouty or rheumatoid arthritis [3, 4, 7]. Common systemic symptoms such as fever, night sweats, and weight loss may be absent [8]. Additionally, inflammatory markers like erythrocyte sedimentation rate (ESR) and C‐reactive protein (CRP) may remain within normal limits [3, 8]. These factors make early diagnosis and treatment challenging. Furthermore, radiological signs appear late in the disease course, and there are no definitive radiological indicators of the condition [9]. Similarly, secondary pyogenic infections may be superimposed on tuberculosis, further complicating the clinical picture. As seen in our patient, multiple courses of antibiotics may be unnecessarily administered due to misdiagnosis or coexisting pyogenic infection [10]. Therefore, a high degree of clinical suspicion is essential for early diagnosis. Delayed treatment increases disease severity and facilitates the spread of infection to adjacent bones and joints [3, 7, 11].

The dissemination of tuberculosis in the foot is particularly concerning because it can spread to the interconnected joints of Chopart and Lisfranc [3, 11]. Smear microscopy and Xpert MTB/RIF assays are useful for rapid diagnosis [12]. However, tissue samples may contain few or no detectable organisms since skeletal tuberculosis is a paucibacillary disease. Thus, diagnosis primarily relies on histopathological findings, such as the presence of epithelioid granulomas with caseous necrosis and giant cells [3, 5]. Based on our experience, tissue biopsy remains the most reliable diagnostic tool for musculoskeletal tuberculosis. Although GeneXpert and PCR offer greater sensitivity than conventional staining methods, a diagnosis of musculoskeletal tuberculosis can still be missed due to its paucibacillary nature. Furthermore, the high sensitivity of GeneXpert and other molecular diagnostic tools can lead to a false sense of exclusion when results are negative, potentially diverting clinical suspicion and complicating the diagnostic process. Therefore, in this case, we opted for tissue biopsy rather than GeneXpert following a negative AFB stain.

Anti‐tubercular therapy (ATT) remains the mainstay of treatment for extrapulmonary tuberculosis, including skeletal tuberculosis [3, 4, 13]. First‐line anti‐tubercular drugs include isoniazid, rifampicin, pyrazinamide, and ethambutol. The treatment regimen should be adjusted based on drug susceptibility testing [14].

Surgical intervention is often required in cases of skeletal tuberculosis [6]. Surgery may be indicated for various reasons, including abscess drainage, bone curettage, tissue biopsy, stabilization of bones and joints, or deformity correction. Early debridement reduces the bacterial load and enhances vascularity in the affected region, both of which increase the penetration of anti‐tubercular drugs, resulting in a better prognosis [15]. Due to the rarity of foot tuberculosis, literature on this subject is limited. Zou et al. [16] reported a two‐stage surgical approach for treating midfoot tuberculosis, with a median bone union time of 3.8 months. Immobilization of the affected joints is crucial to prevent deformities [13].

A two‐stage surgical approach has been demonstrated to be successful by many authors for midfoot tuberculosis [13, 16, 17]. Fusion rods have also been used for stabilization of the affected joint [17]. Recovery with anti‐tubercular therapy alone, without the need for surgery, has also been reported [15, 18].

In this case report, we opted for a two‐stage surgical approach. Clinical history, examination, and investigations pointed to a chronic infectious etiology. Pus culture revealed *Staphylococcus aureus* , prompting the administration of appropriate intravenous culture‐sensitive antibiotics to control the infection. In the first stage, saucerization of the affected bone was performed to remove infected tissue and bone, followed by implantation of vancomycin‐coated bone cement. In the second stage, the bone cement was replaced with a tricortical iliac bone graft, which was fixed using a Kirschner wire. Complete bone union was achieved within three months.

There is a lack of research on a one‐stage surgical approach for foot tuberculosis. Given the increasing incidence of tuberculosis, further studies should explore whether a one‐stage procedure is as effective as the traditional two‐stage approach [17]. Additionally, standardized medical management for skeletal tuberculosis is lacking, unlike the well‐established regimen for pulmonary tuberculosis. More research is needed to determine the frequency of co‐existing tuberculous and pyogenic osteomyelitis, as observed in this case. It is also crucial to investigate whether the presence of pyogenic infection necessitates modifications in surgical or medical treatment strategies.

## Author Contributions

**Dahal Sweekar:** supervision, writing – original draft, writing – review and editing. **Bist Omkar:** writing – original draft, writing – review and editing. **Bhatta Sunil:** writing – review and editing. **Khati Sriram:** writing – review and editing. **Upadhyaya Maya:** writing – review and editing. **Pant Shubham:** writing – review and editing.

## Ethics Statement

The authors have nothing to report.

## Consent

Written informed consent was obtained from the patient for the publication of any potentially identifiable images or data included in this article.

## Conflicts of Interest

The authors declare no conflicts of interest.

## Data Availability

The raw data supporting the conclusions of this article will be made available by the authors without undue reservation.
